# Immune Responses Potentially Involved in the Gestational Complications of *Brucella* Infection

**DOI:** 10.3390/pathogens12121450

**Published:** 2023-12-14

**Authors:** Lucía Zavattieri, Florencia Muñoz González, Mariana C. Ferrero, Pablo C. Baldi

**Affiliations:** 1 Cátedra de Inmunología, Facultad de Farmacia y Bioquímica, Universidad de Buenos Aires, Buenos Aires 1113, Argentina; mv.lzavattieri@gmail.com (L.Z.); flor.mg@live.com.ar (F.M.G.); ferrerom@ffyb.uba.ar (M.C.F.); 2 Instituto de Estudios de la Inmunidad Humoral (IDEHU), Consejo Nacional de Investigaciones Científicas y Técnicas (CONICET), Universidad de Buenos Aires, Buenos Aires 1113, Argentina

**Keywords:** *Brucella*, placentitis, abortion, vertical transmission, inflammation, trophoblasts, intracellular replication, endometrial cells, decidua

## Abstract

Infection by *Brucella* species in pregnant animals and humans is associated with an increased risk of abortion, preterm birth, and transmission of the infection to the offspring. The pathogen has a marked tropism for the placenta and the pregnant uterus and has the ability to invade and replicate within cells of the maternal–fetal unit, including trophoblasts and decidual cells. Placentitis is a common finding in infected pregnant animals. Several proinflammatory factors have been found to be increased in both the placenta of *Brucella*-infected animals and in trophoblasts or decidual cells infected in vitro. As normal pregnancies require an anti-inflammatory placental environment during most of the gestational period, *Brucella*-induced placentitis is thought to be associated with the obstetric complications of brucellosis. A few studies suggest that the blockade of proinflammatory factors may prevent abortion in these cases.

## 1. Introduction

Brucellosis is a worldwide-distributed infectious disease caused by several species of Gram-negative bacteria of the genus *Brucella*, which primarily affects domestic animals and wildlife, from which it is transmitted to humans. *B. melitensis*, *B. suis*, and *B. abortus* are the most pathogenic species for humans, and each one has a domestic animal as a preferential host (small ruminants, swine, and bovines, respectively). Brucellosis has a significant impact on public health and is considered the most common zoonosis [1]. Recent reports suggest at least 1.6–2.1 million new cases of human brucellosis every year [2]. A distinctive trait of most *Brucella* species is their capacity to invade, replicate, and survive efficiently in phagocytic and several non-phagocytic cells, which explains their tendency to produce chronic disease. Brucellosis is a debilitating but rarely fatal disease. In humans, the acute phase is usually accompanied by nonspecific findings such as fever, sweats, splenomegaly, weight loss, myalgia, and arthralgia, whereas the chronic disease can present complications such as osteoarticular brucellosis, neurobrucellosis, and endocarditis [3].

In animals, one of the best-documented characteristics of brucellosis is its tendency to produce reproductive diseases, including abortions, preterm birth, orchitis, epididymitis, and infertility. In contrast, there have been controversial data regarding the relationship between brucellosis and pregnancy outcomes in humans [4]. While older studies suggested that the disease does not affect human pregnancy, some contemporary studies indicate that brucellosis leads to adverse obstetric outcomes in humans, including a higher abortion rate than that associated with other bacterial pathogens. Of note, the pathogen has been isolated from human placentas or aborted fetuses. The pathophysiological basis of abortion and other pregnancy complications of brucellosis has not been defined, but it is interesting to note that placentitis has been described in many studies on *Brucella*-related abortion in animals. It is well established that a successful gestation requires the maintenance of an anti-inflammatory environment in the maternal–fetal unit during most of the pregnancy period, except for the initial phase to promote implantation and the final phase for labor induction, and that events leading to inflammation phenomena during the intermediate phase are associated with different adverse outcomes, including abortion and preterm birth [5]. From the standpoint of adaptive immunity, a healthy pregnancy is favored by the maintenance, in most of the gestational period, of a balance favoring Th2 and Treg responses against Th1 and Th17 profiles. Given the known relationship between placentitis and adverse pregnancy outcomes in several gestational infections [6], a role for placental inflammation in *Brucella*-induced abortion and preterm birth has been suggested [7,8,9]. While the inflammatory response of trophoblasts to *Brucella* has been recently reviewed [10], here we review epidemiological, clinical, and pathological aspects of *Brucella*-induced pregnancy complications and analyze the ability of the pathogen to infect and replicate in different cell types of the maternal–fetal unit and induce inflammatory responses that may lead to such complications.

## 2. Epidemiology of *Brucella*-Induced Pregnancy Complications

In their preferential hosts, *Brucella* spp. mainly affect the reproductive tract. In bovine and caprine brucellosis, abortion is one of the most characteristic clinical signs, which occurs in the middle to late stages of gestation [11] at a rate that varies from 30 to 80% in susceptible herds. Also, in dogs, the classic symptom of *B. canis* infection is late abortion, between 30 and 57 days of gestation, with a higher frequency noted between days 45 and 55 [12].

On the other hand, in swine brucellosis, abortion is generally a minor disease presentation under field conditions, as infection could result in small fetuses expelled in placental tissues that are rarely detected by farmers. The first evidence of early abortions may be a return to estrus at 40–45 days after natural breeding [13]. Regarding *B. ovis* infection, it rarely causes abortions in ewes and rarely extends from one pregnancy to the next [14,15] (Table 1).

Although abortion in animals caused by *Brucella* infection is well known, for several years, there has been controversy about the relationship between human brucellosis and pregnancy outcomes. In pregnant women, the seroprevalence of brucellosis varies from 1.3 to 12.2% [16,17,18,19,20]. Such variability depends on livestock contact, as pregnant women without animal-related occupations show a lower seroprevalence of brucellosis [21] than women from agro-pastoral communities, who do most of the work associated with the care and harvest of livestock products [22]. In the last decades, there have been more reports of adverse outcomes in *Brucella*-infected pregnant women [17,23]. Spontaneous miscarriage rates range from 18.6 to 73.3% [16,24]. Potential factors affecting the rate of miscarriage include the infecting species (*B. melitensis* is usually regarded as more virulent), the infection route (food versus other sources), and the median age of the mothers. Interestingly, most of the cases are documented to occur during the first and second trimesters of gestation and differ from the time of abortion occurrence in animals, commonly manifested at later gestational stages [25]. Other adverse pregnancy outcomes related to *Brucella* infection have been described, including preterm birth [17,18,26,27,28,29], intrauterine fetal death (IUFD) [17], and neonatal or maternal death [4] (Table 1). Differences in obstetric complication rates and maternal or fetal mortality may be attributed to early diagnosis and treatment of brucellosis in different countries [30].

The correlation between bacteraemia or antibody titers and the incidence of obstetrical complications is not clear. Some authors support that high antibody titers or bacteraemia are associated with abortion occurrence [17,23,31], while others have failed to show this association [16,32].

Regarding the prevalence of *Brucella* species among pregnant women, *B. melitensis* is known to be responsible for the majority of cases, recurrences, and chronic stages in the world [1,30,33,34]. However, in some regions, *B. abortus* may be the prevalent species among women with spontaneous abortion [35]. Also, *B. suis* has been occasionally reported to cause abortion in humans [36]. The increased frequency of associations between *B. melitensis* and abortions in pregnant women within endemic regions, when compared to *B. suis* or *B. abortus*, may be attributed to either its heightened virulence or the elevated consumption of fresh goat milk products in those endemic areas. Conducting a global epidemiological study is essential to determine whether there is a heightened occurrence of abortions in women infected with *B. melitensis* as opposed to those infected with *B. abortus* or *B. suis*.

*Brucella* infection during pregnancy can not only induce abortion and preterm birth but may also lead to vertical transmission of the pathogen (Table 1). Transmission of *Brucella* from the mother to the offspring may occur before birth (congenital) or after birth (neonatal). Congenital brucellosis occurs through transplacental transmission, whereas neonatal brucellosis may be acquired by contact with body fluids secreted during delivery or through breastfeeding [4]. Placental transmission of *Brucella* to the fetus has been widely described in animals but only rarely in humans. *Brucella melitensis* is the species most frequently associated with vertical transmission in humans, although cases due to *B. abortus* have also been reported [37]. In dogs, in contrast, *B. canis* and *B. suis* have been implicated, and in the first case, bacteria have been found in several organs from aborted fetuses or infected newborns [38]. The consequences of the infection for the neonate are diverse and may include respiratory distress syndrome, sepsis, and multiple organ failure, with high rates of morbidity and mortality [4]. Notably, congenital brucellosis may impact the reproductive capacity of the offspring. Animals born to an infected pregnant mouse will have a reduced rate of pregnancy and reduced birth weight compared to those born to a healthy mother. A study showed that the number of fetuses born to the “infected” first generation (mice born to an infected mother) was about half compared to those produced by an uninfected first generation [39]. However, it must be kept in mind that the mouse is just an experimental model (see below) and does not constitute a natural host for any known *Brucella* species.

## 3. *Brucella* Vaccines and Gestational Complications in Animals

In many developing countries, brucellosis leads to substantial economic losses due to abortions and infertility in pregnant livestock. To prevent infections and abortions, which not only result in economic setbacks but also contribute to the spread of the bacterium within herds and pose a risk of human infection, it is recommended to vaccinate animals alongside implementing testing and slaughter measures. Currently, licensed available vaccines include live attenuated strains, such as *B. abortus* S19 (S19) and *B. abortus* RB51 (RB51) for bovines and *B. melitensis* Rev.1 (Rev.1) for small ruminants. There are no vaccines available for dogs and pigs. The Rev. 1 strain is widely used worldwide. When administered to sexually immature females, the vaccine is safe and induces long-lasting protection against *B. melitensis* infection and abortion [40]. However, administering Rev. 1 during gestation results in a variable abortion rate of 40% to 80%, which can spread disease within the herd and pose a risk to individuals handling aborted placentas and fetuses, as this vaccine strain can cause disease in humans [40,41,42,43]. *B. abortus* S19 is a naturally attenuated strain with a deletion in the erythritol catabolic genes [44], whereas RB51 is a rough mutant strain derived from *B. abortus* 2308, which lacks the *wboA* gene encoding a glycosyl transferase necessary for O-side chain synthesis [45]. Although numerous studies have shown that S19 and RB51 vaccinations protect approximately 65–75% of cows against abortion and infection [46], their administration during pregnancy can cause abortion in cows. Additionally, despite being attenuated in animals, both vaccine strains are infectious to humans [47]. Due to the drawbacks associated with the vaccines mentioned above, several studies have been directed toward the development of new vaccines that are both safe and effective. One strategy involves the creation of subunit vaccines derived from *Brucella* (including lipopolysaccharides, proteins, DNA, and outer membrane vesicles). Another approach includes the use of new live attenuated mutant strains capable of protecting against virulent *Brucella* infection, yet without the adverse effects associated with commercial attenuated vaccines [43]. Despite the induction of abortions in pregnant animals being one of the primary adverse effects of live attenuated vaccines, only a limited number of studies have investigated the efficacy, safety, and vertical transmission of these novel live attenuated vaccines when administered to pregnant animals. Subcutaneous vaccination of pregnant sheep with *B. melitensis* 16MΔvjbR resulted in fewer abortions and less vertical transmission compared to Rev.1. Interestingly, the level of protection was similar between Rev. 1 and 16MΔvjbR [43]. In a study by Zriba et al. [48], the potential use of the commercially available vaccine for cattle or the live attenuated vaccine candidate *B. abortus* S19ΔvjbR to protect swine from brucellosis was investigated. Vaccination with S19 or S19ΔvjbR in pregnant swine did not induce abortion, stillbirths, reduction in litter size, or vertical transmission. Recently, Zabalza-Baranguá et al. [49] evaluated the safety of the *B. melitensis* 16MDwzm in-frame deletion mutant in pregnant mice and sheep. In mice, 16MDwzm prevented placental and vertical transmission before parturition and protected against *B. melitensis* and *Brucella ovis* infections. In pregnant sheep, while 16MDwzm did not induce abortions or fetal death, some ewes exhibited a transient reactivation of the infection in placentas and/or milk at parturition. These results suggest that the use of this potential vaccine should be avoided during gestation to prevent the dissemination of the vaccine strain during childbirth. The contradictory results regarding the safety of the vaccine in the murine and ovine models underscore the need to evaluate new live attenuated vaccines in natural *Brucella* hosts during pregnancy.

## 4. Pathological Findings in the Infected Placenta

*Brucella* is known to invade and colonize the placenta of both wildlife and domestic animals, with similar pathological findings in all cases [50,51,52,53,54,55]. Since the early studies by Payne [56,57] until the present, different animal models have been tested to explain the pathology of abortion linked to *Brucella* infections, which allowed for reaching a consensus regarding the histopathological findings in the placentas of the different animal species. The most frequent lesions include placentitis, inflammatory infiltrates (including polymorphonuclear cells, lymphocytes, and macrophages), vasculitis, necrosis, and ulcerated or compromised chorioallantoic membrane [58,59,60,61,62,63,64,65,66]. Other more sporadic findings are placental calcifications (associated with chronicity), purulent or fibrinous exudates, granulomas, and placental edema. Placental lesions found in animal models of *Brucella* infection are in line with those found in natural infections. However, the inflammation induced by *B. abortus* in the murine model is much weaker than the severe inflammation seen in the natural host [67]. In contrast, *B. melitensis* produces the same type of lesions in mice, sheep, and goats [68]. Whatever the case, the mouse is widely used as a model of abortion and placentitis induced by different *Brucella* strains [69]. Nevertheless, the most desired models are those that resemble the natural infection regarding host type, placentation type, gestation length, etc. Guinea pigs are preferred as a model when the aim is to elucidate the mechanisms of abortion in women, as they have a discoidal and hemochorial placenta, a gestation period longer than mice, and systemic manifestations similar to human brucellosis. In addition, guinea pigs are susceptible to the most relevant *Brucella* species (*B. suis, B. melitensis,* and *B. abortus*), *B. neotomae*, and *B. ovis*. Non-human primates such as *Macaca mulata* are an excellent model as they share with humans not only the characteristics described but also similar symptomatology.

## 5. *Brucella* Infection and Replication in Placental Cells

Placental infection can originate via two routes: one via sexual transmission, where the pathogen ascends through the genital tract to the placenta, and the other via maternal blood. In humans, there are two points of contact between mother and fetus that could allow transmission of infection: (a) the maternal decidua cells (immune and stroma cells) that come into contact with the extravillous trophoblast (EVT) at the site of implantation and (b) the maternal blood surrounding the syncytiotrophoblast (SYN) [70].

In the face of pathogen entry, there are four main barriers that prevent fetal infection: (1) immune cells present at the maternal–fetal interface, originating from maternal blood, (2) the SYN, a monolayer of multinucleated fused trophoblasts that have intrinsic resistance to infection by certain pathogens, (3) the EVT, which has innate defense mechanisms against pathogen invasion, although it is more susceptible than the SYN, and (4) the basal membrane beneath the trophoblast, representing the last barrier preventing colonization of fetal stroma [70].

The intracellular replication capability of *Brucella* is a fundamental determinant of its pathogenicity both in general and in gestational complications in particular. Within both phagocytic and non-phagocytic cells, the *Brucella*-containing vacuole (BCV) engages in transient interactions with early endosomes, late endosomes, and lysosomes [71]. During this phase, BCVs are identified by lysosomal membrane-associated protein 1 (LAMP1). The acidification of BCVs is essential as it promotes the intracellular expression of genes responsible for encoding the VirB type IV secretion system (T4SS). Subsequently, *Brucella* orchestrates fusion with endoplasmic reticulum (ER) membranes in a VirB T4SS-dependent manner and replicates within ER-derived compartments in both professional and non-professional phagocytes [72,73]. Furthermore, in the advanced stages of infection, the transition from replicative BCVs (rBCVs) to autophagic BCVs (aBCVs) facilitates the release of bacteria from infected cells. The formation of aBCVs necessitates the involvement of autophagy initiation proteins, including ULK1, Beclin 1, and ATG14L, and the activation of PI3-kinase. *Brucella* adeptly exploits this mechanism for intercellular spread [74,75,76]. More recently, it has been found that *B. abortus* also induces a mitophagy pathway required for the completion of its intracellular cycle and egress from infected HeLa cells and macrophages. This pathway depends on the mitophagy receptor BNIP3L, and depletion of this receptor leads to a reduction in the number of aBCVs in the host cell and the number of bacteria in culture supernatants [77].

The three main zoonotic species, *B. melitensis*, *B. abortus,* and *B. suis*, are able to infect and replicate in human cell lines of cytotrophoblast (CTB) (BeWo and JAR) and EVT (HTR8/SVNeo, JEG-3 and Swan-71) in vitro [8,68,69] (Table 2). In CTB, *B. abortus* and *B. suis* replicate through the formation of their conventional BCVs. In contrast, in the EVT cell line JEG-3, both strains replicate by forming inclusions in a different vacuole (LAMP1+ and calnexin-). Such replication is not fully T4SS-dependent, whereas *B. melitensis* replicates in these cells in conventional BCVs in a VirB-dependent manner. These findings are consistent with those observed in trophoblasts isolated from human placentas at term where *B. abortus* is able to replicate both in CTB (in conventional BCVs) and EVT (also forming inclusions) [78]. In contrast to what was observed in JEG 3, *B. abortus* replicates in primary trophoblasts and EVTs of the Swan-71 cell line in a VirB-dependent manner [8,78,79]. In addition, *B. papionis* (a species associated with gestational complications in primates) and *B. melitensis* replicate efficiently in SYN (fused BeWo cells), which may give *Brucella* the ability to cross the epithelial barrier and infect the fetus. Interestingly, *B. papionis* replicates in human CTB or SYN but not in EVT. However, it is capable of transmitting an active infection from EVT to CTB [79].

Hormone secretion is essential for placental development. In vitro infection with *B. abortus* and *B. melitensis* does not affect human chorionic gonadotrophin secretion in JEG-3 cells. However, *B. melitensis* infection decreases progesterone and estradiol production in these cells [78,79]. Consistent with these results, *B. abortus* infection has been shown to suppress placental progesterone production in the mouse pregnancy model [9].

In hemochorial placentation (humans, mice), the invasion of the maternal endometrium by EVT typically occurs in the early stages of pregnancy and is a critical step in anchoring the placenta. Furthermore, as the transition from the first to the second trimester occurs, EVT plays an additional role in remodeling uterine arteries, facilitating maternal blood flow into the placental intervillous space. This, in turn, ensures the delivery of essential nutrients and oxygen to the developing fetus. It has been observed that infection with *B. melitensis*, but not *B. suis* or *B. abortus,* diminishes the invasiveness of JEG-3 cells [78]. This reduction in invasiveness could potentially impact implantation and the adequate supply of nutrients and oxygen to the developing fetus. The failure of trophoblast functionality is not due to a cytotoxic effect of *Brucella* since infection with virulent species did not affect human trophoblast viability [8,78,79]. Of note, however, *B. melitensis* infection of JEG-3 cells increased the expression levels of CD98hc, a protein involved in the regulation of integrin-mediated signaling, and the authors hypothesized that this change may have a role in the reduced invasiveness of the infected EVT [79]. In contrast, the infection with either *B. melitensis* or *B. papionis* reduced CD98hc expression in BeWo cells (CTB). Interestingly, *B. papionis* infection reduced the ability of these cells to form SYN.

As mentioned, *B. abortus* infects placental trophoblast from experimentally inoculated cows and goats [57,80]. Histological analyses of these tissues revealed that *Brucella* replicates within intracellular compartments closely associated with the rough ER and shows evidence of cell death [58,61]. It has also been shown that *B. abortus* can infect and grow within trophoblasts of bovine chorioallantoic membrane (CAM) explants obtained from seven-month-old gravid cows but fails to grow within CAM obtained from three-month-old gravid cows [81]. Consistent with these results, it was demonstrated that *B. abortus* infects bovine trophoblast cell lines in different stages of placental growth [82]. However, efficient replication occurs only in cell lines from late gestational periods. This suggests that while the bacteria may infect the trophoblast early in gestation, their substantial multiplication occurs only when specific intracellular cellular conditions are met during later gestational stages.

The reasons for the selective colonization of ruminant placenta by *Brucella* are unknown [81,83]. Erythritol is the main carbon source for most *Brucella* species. It has long been assumed that its abundance in the placenta of ruminants and pigs could at least partly explain the characteristic genital tropism observed in *Brucella* [84]. Recently, Barbier et al. [85] evaluated the availability and indispensability of erythritol during intracellular multiplication of *Brucella* in human and rodent cells. They studied the replication properties of *B. abortus* mutants for different enzymes of the erythritol catabolism pathway, *B. abortus* Δ*eryH* (erythritol-sensitive) and Δ*eryA* (erythritol-tolerant but with reduced growth when erythritol is a vital nutrient). The evaluation was carried out in various infection models, revealing that despite the presence of erythritol in human, bovine, and murine trophoblasts, its availability was not essential for *B. abortus* multiplication. However, the trophoblast and decidua of *B. abortus*-infected placental mice showed the presence of aldose reductase, an enzyme capable of generating erythritol from precursors in the pentose cycle [85]. Further research is imperative to elucidate the precise role of aldose reductase in tropism and inflammation, particularly in the context of *Brucella*-induced abortion.

In contrast to human trophoblast cell lines, *Brucella* infection in pregnant goats, cows, and mice induces the death of infected trophoblasts. However, the mechanisms involved in trophoblast death are not fully understood. Results from infection in the murine pregnancy model showed that trophoblast death induced by *B. abortus* depends on T4SS and on the T4SS effector VceC. This later protein triggers an ER stress response involving the transcription factor CHOP [86]. Although NOD1/NOD2 expression in *Brucella*-infected macrophages contributed to ER stress-induced inflammation, NOD1/NOD2 knockout mice were not completely resistant to *B. abortus*-induced abortion, indicating the existence of other cellular pathways involved in triggering trophoblast death [67]. Another T4SS effector, VceA, seems to modulate autophagy and apoptosis in trophoblasts. Autophagy markers were increased, and the expression of apoptosis-related genes was decreased in the HPT-8 trophoblastic cell line infected with a VceA deletion mutant as compared to infection with the wild-type strain [87].

As mentioned above, for pathogens that reach the placenta through the bloodstream, the maternal decidua represents the site of initial placental colonization. We have recently demonstrated that *B. abortus* efficiently infects and replicates in human decidualized endometrial cells. *Brucella* replication in these cells was T4SS-dependent [88]. It remains to be determined whether infected decidualized cells can transmit the infection to EVT at the contact site during implantation.

## 6. *Brucella*-Induced Inflammatory Responses in Trophoblasts and Other Cells from the Maternal—Fetal Unit

As mentioned above, several studies have shown that *Brucella* infection induces placentitis in pregnant animals. In addition, histological analyses indicate that trophoblasts are a central target of the pathogen, and studies performed with trophoblastic cell lines revealed the ability of *Brucella* to manipulate cellular mechanisms to promote its survival and replication in these cells. Of note, several studies have suggested that infected trophoblasts may have an important role in the *Brucella*-induced inflammatory phenomena in the placenta. Fernandez et al. [8] showed that *B. abortus* infection significantly increases the production of interleukin 8 (IL-8), monocyte chemotactic protein 1 (MCP-1), GM-CSF, and IL-6 in the human trophoblastic cell line Swan-71. Taking into account that, during *Brucella* infections, placental trophoblasts could interact with decidual macrophages or with monocytes and neutrophils attracted to the infection site by chemokines, the authors also analyzed the production of proinflammatory factors in the context of the interaction of trophoblasts with infected phagocytes. Of note, the stimulation of Swan-71 cells with conditioned medium (CM) from *B. abortus*-infected human monocytes (THP-1 cells), macrophages, or neutrophils induced a significant increase of IL-8, MCP-1, and IL-6 compared to stimulation with CM from non-infected cells. Neutralization studies showed that IL-1β is involved in the stimulating effects of CM from infected phagocytes on the production of the three cytokines by Swan-71 cells, whereas TNF-α is also involved in the induction of MCP-1. Interestingly, a reciprocal stimulating effect was observed. When human monocytes and neutrophils were stimulated with CM from *Brucella*-infected Swan-71 cells, an increased production of IL-8 and/or IL-6 was detected in the phagocytes. Therefore, trophoblasts may contribute to *Brucella*-induced placentitis not only by recruiting phagocytes to the site of infection but also by stimulating these cells to secrete proinflammatory chemokines and cytokines. Globally, cross-talk between trophoblasts and phagocytes may take place during placental infections by *B. abortus* (and probably by other *Brucella* species), leading to increased levels of proinflammatory cytokines.

A similar proinflammatory response has been shown for canine trophoblasts [89]. Primary canine trophoblasts isolated from the placenta of healthy pregnant bitches responded to *B. canis* infection with increased levels of IL-8 and RANTES (CCL5). Similar to the situation with human trophoblasts, the stimulation of canine trophoblasts with CM from *B. canis*-infected monocytes and neutrophils also induced a significant increase of IL-8, IL-6, and RANTES secretion compared to stimulation with control CM. While not formally tested in this study, the fact that TNF-α levels were significantly increased in CM from *B*. *canis*-infected canine neutrophils and monocytes suggests that this factor may be involved in the stimulating effect of phagocytes on trophoblast cytokines. As IL-8 is a chemoattractant for neutrophils and RANTES is a chemoattractant for a variety of leukocytes in inflammatory sites, these results suggest that trophoblasts-derived chemokines may be involved in the development of the neutrophilic and histiocytic infiltrates usually observed in the placentas of *B*. *canis*-induced canine abortions [54,55,90]. Overall, these results suggest that human and canine trophoblasts may contribute to the local inflammatory environment in the placenta during *Brucella* infections either through a direct response to the pathogen or through interactions with infected phagocytes, potentially contributing to the pregnancy complications of brucellosis.

Bovine trophoblasts also respond to *Brucella* infection with an increased production of proinflammatory cytokines. When explants of chroioallantoic membranes obtained from healthy cows were infected on their trophoblastic side with *B. abortus*, a reduced expression of some proinflammatory genes was observed at 4 h post-inoculation by microarray and RT-PCR [62]. However, this seemed to be a transient phenomenon, as the expression of CXCL8 and CXCL6 was significantly increased at 12 h post-infection (last time measured). Of note, these chemokines also exhibited increased gene expression (about 10-fold) in the placentomes of cows experimentally infected with *B. abortus* at 6–7 months of pregnancy, having either normal parturition or abortion. A later study by the same group revealed that the type IV secretion system (T4SS, virB operon) and the TIR-domain containing protein BtpB of *Brucella* are involved in the early downmodulation of proinflammatory genes in the infected chorioallantoic membrane [91].

A few additional studies have been performed to identify bacterial or host factors involved in the inflammatory response of trophoblasts to *Brucella* infection. A study by Liu et al. [92] investigated the role of high-mobility group box 1 (HMGB1) in regulating the inflammatory response of primary murine trophoblasts to *B. melitensis* infection. HMGB1, which is present in all cell types, is a damage-associated molecular pattern (DAMP) and is a known mediator of inflammatory response during sterile and infection-associated diseases. Compared with uninfected cells, the levels of TNF-α, IL-6, RANTES, and IFN-γ were increased in *B. melitensis*-infected murine trophoblasts. Treatment with a neutralizing anti-HMGB1 antibody significantly reduced secretion of TNF-α and IL-6 protein levels but did not modify RANTES or IFN-γ. Similarly, treatment of infected trophoblasts with recombinant HMGB1 increased TNF-α and IL-6 levels but had no effect on RANTES or IFN-γ levels. These data suggest that part of inflammatory cytokines in *Brucella*-infected trophoblasts might result from HMGB1 stimulation. Further studies performed with the HPT-8 cell line of spontaneously immortalized human trophoblasts derived from first-trimester placenta revealed that HMGB1 mediates pro-inflammatory production in *B. melitensis*-infected trophoblasts via activation of MAPK and NF-κB pathways. Of note, HMGB1 released by infected trophoblasts also seems to mediate inflammatory responses in neighboring cells. Stimulation of bone marrow-derived macrophages (BMDMs) with conditioned medium (CM) from infected trophoblasts induced a significant increase in the secretion of TNF-α and IL-6, and this inducing effect was partially abolished by the treatment of CM with anti-HMGB1 neutralizing antibodies. Of note, an increased expression of HMGB1 has also been detected in explants of bovine chorioallantoic membrane infected with *B. abortus* [93].

*Brucella* outer membrane protein Omp25 has been claimed to induce cytokine responses in HPT-8 cells. However, the differences in the levels of the three evaluated cytokines (TNF-α, IL-1β, and IL-10) between cells infected with *B. abortus* S2308 or its ΔOmp25 mutant were quite small. Nevertheless, the mutant induced a markedly weaker stimulation of p38, ERK1/2, and JNK kinases [94].

Besides trophoblasts, endometrial cells may also be relevant for the induction of inflammatory responses in the maternal–fetal unit. When the blastocyst initiates its implantation in the uterus, trophoblasts begin to invade the endometrial epithelium and the underlying stroma. Stromal cells respond by producing the decidual reaction (epithelial transformation of fibroblasts with glycogen and lipids storage), and this endometrial region transforms into the decidua. The decidual stromal cells secrete prolactin, insulin growth factor-binding protein, and several cytokines that regulate innate immunity [95]. The maternal decidua may be the initial site of placental colonization for *Brucella*, as has been described for several microorganisms that reach the placenta by the hematogenous route [70,96]. For this reason, the ability of decidual cells to respond to *Brucella* is especially relevant.

A study by Zavattieri et al. [88] evaluated the ability of *B. abortus* to invade and establish a replicative niche in non-decidualized and decidualized human endometrial stromal cells (T-HESC cell line). *B. abortus* was able to infect T-HESC cells in both conditions, with a slightly higher number of intracellular bacteria for non-decidualized cells at the beginning of the infection. The pathogen was able to replicate inside decidualized and non-decidualized T-HESC cells, but this ability was lost in a mutant lacking a functional virB operon. The production of prolactin by infected decidualized T-HESC did not differ from that of uninfected controls, showing that *B. abortus* infection does not affect the decidualization status of the cells. Both decidualized and non-decidualized cells increased their production of CXCL-8 (IL-8) and MCP-1 in response to infection with either wild-type *B. abortus* or a double mutant for the BtpA and BtpB proteins involved in TLR signaling modulation. At 48 h post-infection., the levels of IL-8 were higher in non-decidualized cells, but no difference was found for MCP-1 levels. Heat-killed *B. abortus* and outer membrane vesicles released by viable bacteria also induced the secretion of one or both chemokines by decidualized T-HESC, albeit at levels lower than those induced by the infection. Considering that, in the context of *Brucella* infection in the pregnant uterus, endometrial cells may be stimulated not only by the pathogen but also by factors secreted by adjacent infected macrophages, the cytokine response of decidualized T-HESC cells to stimulation with conditioned media from *B. abortus*-infected macrophages was analyzed. Such stimulation induced significant production of IL-6, MCP-1, and IL-8 by T-HESC cells. Additional experiments revealed that IL-1β and TNF-α are involved in the stimulation of IL-6 production, whereas only TNF-α seems to induce MCP-1 and IL-8 secretion. Globally, the results suggest that during *B. abortus* infection in pregnant females, endometrial cells may produce proinflammatory factors not only in response to bacterial antigens but also to stimulation by factors produced by adjacent *Brucella*-infected macrophages. These proinflammatory responses and cellular interactions may be long-lasting due to the ability of *Brucella* to survive and replicate in macrophages and endometrial cells and may contribute to the gestational complications of brucellosis.

In addition to the studies described above, which reveal a potential role for cells of the maternal–fetal unit in the production of proinflammatory factors in response to *Brucella* infection, other studies have focused on the association between systemic levels of proinflammatory molecules and *Brucella*-induced abortion. A study by Kim et al. [97] using the mouse model of *B. abortus* infection during pregnancy found high rates of abortion when the infection was performed on day 4.5 of gestation. A high degree of bacterial colonization was observed in the placenta, and many bacteria were detected in trophoblast giant cells. Serum levels of IFN-γ were increased during the first days of *Brucella* infection (peak at 3 days) in both non-pregnant and pregnant mice, but in the latter, they were associated with abortion as demonstrated by the complete prevention of abortion when pregnant mice were pretreated with a neutralizing antibody against IFN-γ one day before infection. This study did not establish whether IFN-γ production is also increased locally in the uterus or the placenta.

Using the same model of infection in pregnant mice, the same research group revealed that RANTES plays an important role in *B. abortus*-induced abortion [98]. The levels of this cytokine were increased within 3 days of infection in pregnant mice, and pretreatment with an anti-RANTES neutralizing antibody one day before infection prevented *Brucella*-induced abortions. As in the first study, the local production of RANTES was not evaluated in the placenta.

As mentioned above, *B. abortus* infection has been shown to suppress placental progesterone production in the mouse pregnancy model, which is accompanied by reduced serum levels of this hormone [9]. An interesting finding of this study was the inverse relationship between progesterone and proinflammatory factors in these animals. The administration of progesterone at day 1 and 2 post-infection decreased the expression of IFN-γ and IL-6 in the spleen of the infected pregnant mice, and the same happened with the serum levels of these cytokines. Similarly, progesterone reduced TNF-α and IL-6 production by primary mouse trophoblasts infected with *B. abortus* and increased IL-10 production in these cells. Progesterone injection in infected pregnant mice reduced IFN-γ and IL-6 transcripts in the placenta, diminished the severity of placentitis, and increased the number of viable pups. These results suggest that the reduced production of progesterone in *B. abortus*-infected pregnant animals may contribute to abortion through increased expression of proinflammatory factors in the placenta.

## 7. Conclusions

Abortions associated with *Brucella* infections have been well documented, not only in domestic animals but also in wildlife, and there is currently a wide consensus regarding the relationship between brucellosis and gestational complications in humans. In many cases, the association with human abortions derives from serological studies in cohorts of pregnant women, but in selected cases, the pathogen has been isolated from placental and fetal tissues, thus confirming the link between the infection and the obstetric complications. While *B. melitensis* is known to be responsible for the majority of human cases of abortion linked to brucellosis, *B. abortus* has also been identified as the causative agent in some cases.

**Figure 1:**
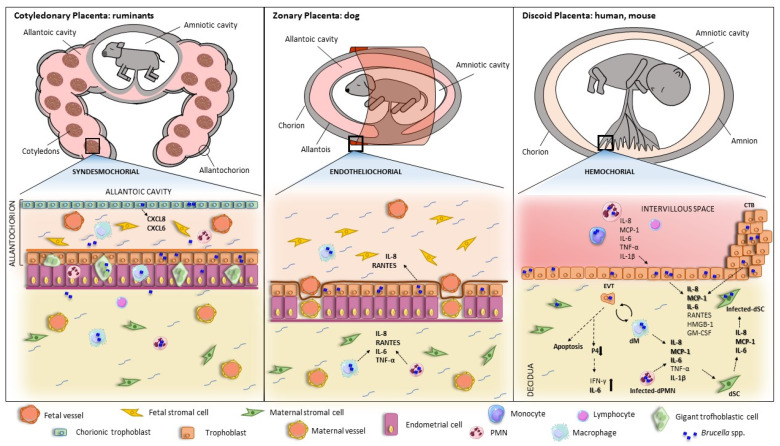
*Brucella* interaction with placental cells in different host species and the resulting inflammatory response. The morphological and histological classification of placentation in different hosts or infection models is depicted. Proinflammatory responses to *Brucella* have been described in the different hosts, although immune responses have been described in greater detail for human lines and the murine model. Cross-talk between trophoblasts and phagocytes takes place during placental infections by *Brucella*, leading to increased levels of proinflammatory cytokines. Infected placental trophoblasts secrete HMGB1, IL-8, MCP-1, GM-CSF, and IL-6, which could attract and activate decidual macrophages, monocytes, and PMN to the site of infection. Infected monocytes and PMN produce high levels of IL-8, IL-6, MCP-1, TNF-α, and IL-1 β that potentiate EVT proinflammatory response. The pro-inflammatory environment impacts progesterone production, which results in increased IFN-γ and IL-6 production. Altogether, this inflammatory environment may contribute to the gestational complications of brucellosis. EVT: Extravillous Trophoblast; CTB: Cytotrophoblast; dSC: decidualized Stromal Cell; PMN: Polymorphonuclear cell; dPMN: decidual Polymorphonuclear cell; P4: Progesterone.

Placental inflammation has been a common finding in affected animals and probably explains most of the pathology in *Brucella*-induced abortion and preterm birth, as a successful gestation requires the maintenance of an anti-inflammatory environment in the maternal–fetal unit during most of the pregnancy period. Several studies have shown the ability of different *Brucella* strains to invade and replicate in human and animal trophoblasts, and in vitro studies have shown that these cells produce a wide array of proinflammatory factors in response to the infection, including TNF-α, IL-6, RANTES, MCP-1, and IL-8. These cytokines may mediate several processes that are deleterious for pregnancy (Figure 1), including the infiltration of neutrophils and macrophages (with increased production of reactive oxygen species, proteases, and other harmful products), the induction of matrix metalloproteinases, and the alteration of the hormonal balance required to support gestation (e.g., decreased production of prolactin and hCG [99]).

The understanding of the pathological processes behind *Brucella*-induced gestational complications may help to design preventive therapies but may also increase awareness regarding the link between brucellosis and abortion or preterm birth in humans. Of note, the reproductive consequences of *Brucella* infection are not limited to those occurring during gestation, as it has been known for a long time that, at least in domestic animals, a previous infection may compromise fertility. In this sense, a recent study has shown that *B. abortus* can establish long-lasting infections in the non-gravid uterus in mice, where it induces inflammatory changes. Notably, the chronically infected mice had a significant reduction in the number of pregnancies compared to controls [100].

In summary, both the pathological studies in naturally or experimentally infected animals and the in vitro ones using human cells strongly suggest that placental inflammation may be involved in the adverse reproductive consequences of *Brucella* infection.

The best approach to reduce the rate of *Brucella*-induced reproductive complications in pregnant women is to systematically test for a potential *Brucella* infection at the beginning of pregnancy. Those having evidence of brucellosis should receive appropriate antibiotic therapy. Additionally, pregnant women (especially those without evidence of an ongoing brucellosis) should receive counseling about risk factors for acquiring the disease. This is especially important for women living in endemic areas. Antibiotics constitute the mainstay of treatment for pregnant women in which brucellosis is diagnosed. Several studies have shown that timely antimicrobial therapy reduces the rate of abortion, preterm birth, and congenital brucellosis [16,23]. Whether the addition of anti-inflammatory treatments can help reduce the rate of pregnancy complications linked to *Brucella* infection is currently unknown, but it is interesting to note that experimental studies in animal models of intra-amniotic streptococcal infection have shown a reduction of preterm delivery when anti-inflammatory agents are added to the antimicrobial therapy [101]. The usefulness of this approach in the case of *Brucella* infection remains to be explored.

## Figures and Tables

**Table 1:** Reproductive complications of brucellosis in different species.

*Brucella* Species	Hosts	Gestational Manifestations	Vertical Transmission	Contagion Source
*B. melitensis*	Small ruminants	Abortion, weak offspring, reduced milk yields	+	Contaminated placenta or aborted fetus. Milk
*B. abortus*	Bovines	Abortion, weak offspring, reduced milk yields	+	Contaminated placenta or aborted fetus. Milk
*B. suis* (biovars 1, 2, 3)	Swine	Abortion, weak offspring	+	Contaminated placenta or aborted fetus. Milk. Contaminated semen
*B. canis*	Canines	Abortion, weak offspring	+	Contaminated placenta or aborted fetus. Milk. Contaminated semen
*B. ovis*	Sheep	Abortion, weak offspring (rare)	Not reported	Close contact or mating with infected rams.
*B. melitensis*, *B. abortus*, *B. suis*	Humans	Abortion, preterm birth, intrauterine fetal death, neonatal or maternal death	+	Contaminated milk and dairy products. Tissues or secretions from infected animals. Contaminated aerosols.

**Table 2:** *Brucella* replication in human trophoblasts.

Cell Type (Line/Primary)	*Brucella* Species	Intracellular Replication	Ref.
CTB (BeWo)	*B. abortus*	Typical BCVs	[68]
CTB (BeWo)	*B. melitensis*, *B. papionis*	VirB-dependent	[69]
CTB (Primary)	*B. abortus*	Typical BCV	[68]
EVT (JEG-3)	*B. abortus*, *B. suis*	LAMP^+^/calnexin^-^ inclusions	[68]
EVT (JEG-3)	*B. melitensis*	Typical BCV, VirB-dependent	[68,69]
EVT (Swan-71)	*B. abortus*	VirB-dependent	[8]
EVT (Primary)	*B. abortus*, *B. suis*	LAMP^+^/calnexin^-^ inclusions	[68]
SYN (Fused BeWo)	*B. melitensis*, *B. papionis*, *B. abortus*	BCV (*B. abortus*)	[68,69]

CTB: cytotrophoblasts; EVT: extravillous trophoblasts; SYN: syncitiotrophoblasts; BCV: Brucella-containing vacuoles.

## Data Availability

No new data were created or analyzed in this study.
